# RASSF4 inhibits cell proliferation and increases drug sensitivity in colorectal cancer through YAP/Bcl‐2 pathway

**DOI:** 10.1111/jcmm.17395

**Published:** 2022-05-25

**Authors:** Yong Han, Xiaotang Zhang, Minmin Guan, Cheng Huo, Chunlin Yu, Bin Hu, Jianjun Cai

**Affiliations:** ^1^ Department of Surgical Oncology The Sinopharm Tongmei General Hospital Datong China; ^2^ Department of General Surgery The Sinopharm Tongmei General Hospital Datong China; ^3^ Department of Pathology The Sinopharm Tongmei General Hospital Datong China

**Keywords:** Bcl‐2, colorectal cancer, RASSF4, YAP

## Abstract

The RASSF family proteins have been implicated in the development of human cancers. To date, the expression pattern and biological significance of RASSF4 in colorectal cancers (CRC) have not been fully investigated. In the current study, we explored expression pattern of RASSF4 in 118 CRC specimens and 30 adjacent ‘normal’ colon tissues by immunohistochemistry. The results showed that RASSF4 was downregulated in CRC tissues compared with adjacent ‘normal’ tissues. RASSF4 downregulation significantly associated with advanced tumour‐node‐metastasis (TNM) stage, T status, positive node status and high Ki‐67 index. Analysis of TCGA dataset also supported RASSF4 downregulation in CRC tissues. Ectopically expressed RASSF4 in LoVo cells inhibited cell growth, colony formation, cell cycle progression and increased the sensitivity to 5‐FU treatment. Annexin V/PI apoptosis assay showed that RASSF4 overexpression increased 5‐FU‐induced apoptosis and downregulated the mitochondrial membrane potential. In addition, Western blot demonstrated that RASSF4 overexpression repressed YAP and Bcl‐2 while upregulating p21 expression. YAP knockdown abolished the role of RASSF4 on Bcl‐2. ChIP assay showed that TEAD4, a major YAP binding transcription factor, could bind to the promoter regions of Bcl‐2. In conclusion, our data showed that RASSF4 was downregulated in human CRC. RASSF4 regulated malignant behaviour through YAP/Bcl‐2 signalling in CRC cells.

## 1 INTRODUCTION

Colorectal cancer (CRC) is one of the most commonly diagnosed cancers and the second most deadly cancer.^1^, ^2^ The large number of CRC cases poses a growing global public health challenge. Although the prospect for CRC therapy is generally good, a rising CRC incidence is also emerging. In addition, the prognosis of CRC patients remains unsatisfactory. Therefore, there is a compelling need for identifying new biomarkers and the underlying mechanisms of its progression.

RASSF4 belongs to the RASSF family proteins which have been reported to interact with Ras protein.^3^, ^4^, ^5^, ^6^ RASSF family proteins are frequently inactivated by mutation or DNA methylation during tumour development.^7^

RASSF4 has been reported to directly interact with and activate K‐Ras in a GTP‐dependent manner. Ectopic expression of RASSF4 induces 293T apoptosis in a Ras‐dependent manner and inhibits tumour cell growth.^8^ Previous studies indicated RASSF4 downregulation in several human cancers. Aberrant loss and promoter methylation of RASSF4 has been found in nasopharyngeal carcinoma.^9^ Decreased RASSF4 expression and aberrant CpG island methylation was also found in gastric cardia adenocarcinoma.^10^ RASSF4 is downregulated during multiple myeloma progression and correlates with a poor prognosis.^11^ In addition, RASSF4 has been reported to be downregulated in non‐small cell lung cancers.^12^

To date, the expression pattern and biological roles of RASSF4 in CRC has not been investigated. In this study, we sought to characterize the expression pattern of RASSF4 protein in clinical CRC specimens. We also examined the biological role of RASSF4 in CRC cell lines.

## 2 MATERIALS AND METHODS

### 2.1 Tissue samples

The study protocol was approved by the institutional reviewer board of the Sinopharm Tongmei General Hospital (2020‐56), and written informed consents were provided. The 118 paraffin‐embedded CRC tissue sections and 30 adjacent ‘normal’ colon tissues (at a distance of >5 cm from the edge of the primary tumour) were obtained from the pathology archives at the Sinopharm Tongmei General Hospital, which no longer required to be maintained.

### 2.2 Immunohistochemistry

Tissue sections were deparaffinized using xylene and rehydrated using graded alcohol. Antigen retrieval was performed using 0.01 M Citrate buffer. The section was treated with hydrogen peroxide to block endogenous peroxide and goat serum to decrease non‐specific staining. Then, tissue slides were washed using TBS and incubated with primary antibody against RASSF4 (1:300 dilution, Proteintech) overnight at 4°C. Elivision Super kit (MaiXin) was used for secondary antibody incubation. After washing with TBS, DAB plus kit (MaiXin) was used to develop immunostaining.

RASSF4 staining was scored according to the immunoreactive score system previously reported.^13^, ^14^ Briefly, staining intensity was assigned as 0 (negative staining), 1 (moderate staining) and 2 (strong staining). Staining percentage was scored as 1 (1%–25%), 2 (26%–50%), 3 (51%–75%) and 4 (76%–100%). We calculate the final score by multiplying the intensity score with the percentage of positive expression. Tissue sections with a final score < 3 were regarded as RASSF4 low/negative expression. Sections with a final scored ≥ 3 were considered as RASSF4 high expression.

### 2.3 Cell culture and transfection

LoVo and HCT‐8 cell lines were obtained from the Cell Bank of Type Culture Collection of Chinese Academy of Sciences (Shanghai, China). Cells were cultured in RPMI‐1640 medium with foetal bovine serum (FBS, 10%) in a saturated humidity incubator. RASSF4 siRNA and negative control siRNA were obtained from Dharmacon. DharmaFECT1 was used for siRNA transfection. The RASSF4 plasmid and the corresponding negative pCMV6 empty vector were from OriGene company and transfected into cells using Lipofectamine 3000. Forty hours after transfection, PCR and Western blot were used to test transfection efficiency.

### 2.4 Western blot

Total protein was extracted using RIPA lysis buffer. Forty microgram protein was loaded to SDS‐PAGE. After separation, proteins were transferred to PVDF membranes and incubated overnight at 4°C with primary antibodies including RASSF4 (1:1000; Proteintech), YAP, p21, Bcl‐2 (1:1000; Cell Signaling Technology) and β‐actin (1:3000; Santa Cruz). After incubation with HRP conjugated secondary antibody (1:3000; Santa Cruz) at 37°C for 2 h, proteins were visualized using Thermo ECL substrate (Thermo), and images were captured using DNR BioImaging System.

### 2.5 CCK‐8 assays

CRC cells (about 2500/per well) were seeded in 96‐well plates and cultured for 5 days. For detection of cell viability, 20 μl of CCK‐8 solution (Cell Counting Kit‐8; Dojindo) was added to each well and incubated for 4 h. The absorbance values at 450 nm were measured using microplate reader.

### 2.6 Colony formation assays

CRC cells (about 1000/per well) were seeded in 6‐cm culture dishes and cultured for about 15 days. Then, the plates were fixed using cold methanol and then stained using Giemsa. Colony numbers were counted under a microscope.

### 2.7 Cell cycle and apoptosis analysis

To analyse cell cycle distribution, CRC cells were fixed using 1% paraformaldehyde, washed with cold PBS, then, cells were incubated with 5 mg/ml propidium iodide (PI) and examined using flow cytometer. For detection of apoptosis, BD Annexin V/FITC kit (BD) was used to stain apoptotic CRC cells, which were detected using flow cytometer.

### 2.8 Mitochondrial membrane potential detection

Mitochondrial membrane potential was measured using JC‐1 staining followed by flow cytometry. Cells were treated with 5 μM JC‐1 staining solution (Abcam) for 30 min. Then, cells were washed twice with PBS and examined for green/red fluorescence using a flow cytometer.

### 2.9 Realtime‐PCR

Total RNA was extracted using RNAiso (TaKaRa) and reverse transcribed into cDNA using PrimeScript RT Master Mix Kit (Takara). Realtime‐PCR was conducted using SYBR Master Mix (Thermo). Target gene was normalized to GAPDH using the 2^−ΔΔct^ method. The primer sequences were as follows:

RASSF4 forward, 5′‐AGTTCGCACTCTACATCGTT‐3′,

RASSF4 reverse, 5′‐CCCATGCAGGATTCTGGAAAT‐3′;

GADPH forward, 5′‐GAAATCCCATCACCATCTTCCAG‐3′,

GADPH reverse, 5′‐GAGCCCCAGCCTTCTCCAT‐3′.

### 2.10 Chromatin immunoprecipitation (ChIP) assay

Chromatin immunoprecipitation (ChIP) assay was performed using the Magna ChIP A/G Assay Kit (Millipore). Briefly, cells were crosslinked with 37% formaldehyde. The DNA/protein complexes were treated using TEAD4 and IgG (Cell Signaling Technology) antibodies and Protein A/G magnetic beads. The precipitated chromatin complexes were purified and de‐crosslinked at 62℃ for 2 h. The precipitated DNA fragments were quantified using PCR analysis. The primers for ChIP were listed as follows:

BCL2 position1 forward, 5′ CGGACTAGGTGTTCAGGTGGA 3′,

BCL2 position1 reverse, 5′ CGCCTACACACACACACGTTG 3′;

BCL2 position2 forward, 5′ CCTGGGCAACATAGCAAAAGC 3′,

BCL2 position2 reverse, 5′ CTGTGCCCTGCCTGACATC 3′.

### 2.11 Statistical analysis

SPSS version 16 was used for all analyses. Student's *t*‐test was carried out to compare data between control and experiment group. The χ^2^ test was used to analyse the relationship between RASSF4 expression and clinicopathologic characteristics. *p *< 0.05 was regarded as statistical significance.

## 3 RESULTS

### 3.1 RASSF4 expression is downregulated in colorectal cancer tissues

**FIGURE 1:**
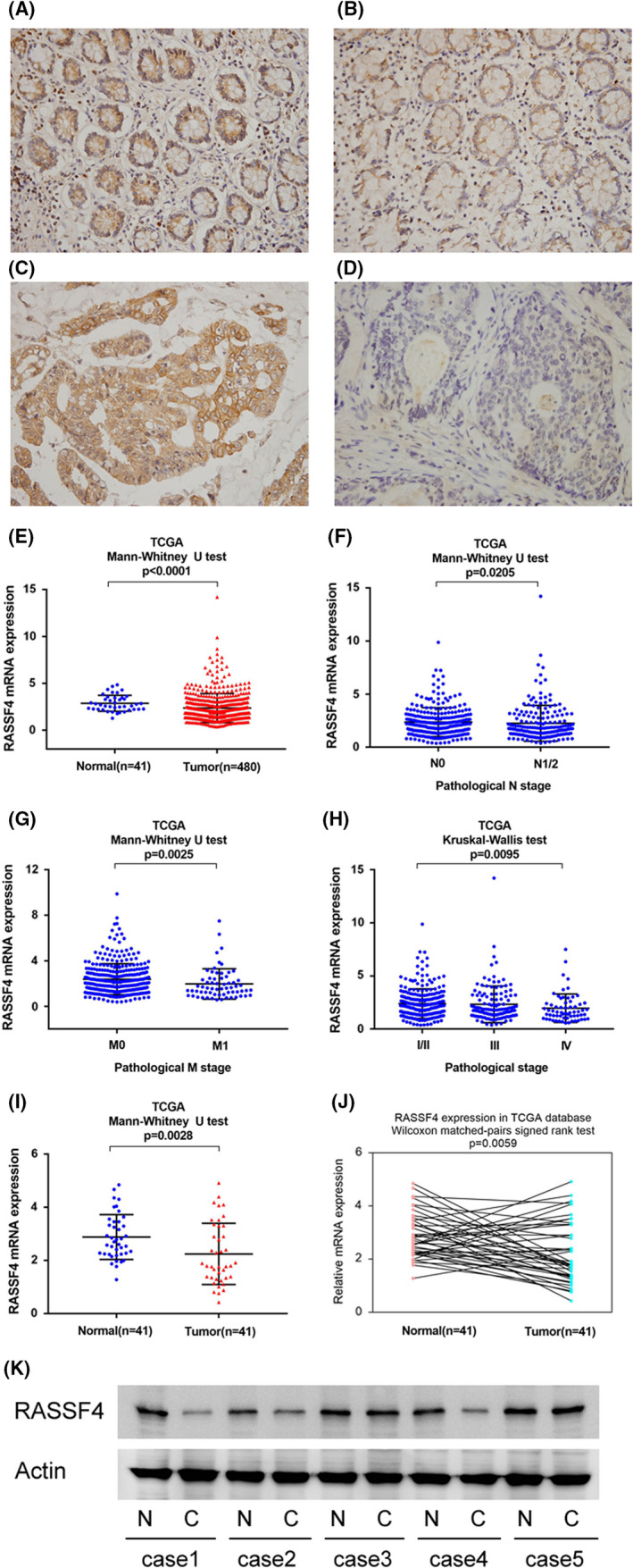
Expression of RASSF4 in colorectal cancer (CRC). (A) Strong RASSF4 expression in normal colon tissue. (B) Moderate RASSF4 expression in a case of normal colon tissue. (C) Strong cytoplasmic RASSF4 expression in a case of CRC. (D) Negative RASSF4 expression in a case of CRC. (E) The Cancer Genome Atlas (TCGA) colorectal cancer cohort analysis. Expression of RASSF4 in CRC tissues was significantly lower than that in normal colon tissues (*p *< 0.0001). (F) TCGA analysis showed that RASSF4 expression was lower in CRC tissues with positive nodal metastasis (*p *= 0.0205). (G) TCGA analysis showed that RASSF4 expression was lower in colorectal cancers with distal metastasis (*p *= 0.0025). (H) TCGA analysis showed that RASSF4 expression was significantly lower in colorectal cancers with higher pathological stage (*p *= 0.0095). (I and J) Analysis of 41 cases of paired tumour/normal samples from TCGA showed that RASSF4 was lower in CRC tissues compared with corresponding adjacent normal colon tissues (*p *< 0.05). (K) Western blot in five colorectal cancer tissues and paired adjacent normal tissues showed RASSF4 downregulation in two out of five of paired cases

We investigated RASSF4 expression in 118 CRC tissue specimens and 30 adjacent ‘normal’ tissue specimens by immunohistochemistry. Strong RASSF4 staining was observed in normal colorectal epithelial tissues (Figure 1A,B). We classified all cases into low or high RASSF4 expression according to staining scores. Statistical analyses revealed that RASSF4 was downregulated in CRC tissues compared with adjacent ‘normal’ tissues (Table 1). Of the 118 cases examined, 73(61.8%) showed high RASSF4 expression (Figure 1C) and 45 (38.2%) exhibited relatively low RASSF4 levels (Figure 1D). Significant correlations were found between RASSF4 low expression status and advanced TNM stage (*p *= 0.0076), high T status (*p *= 0.0036), nodal metastasis (*p *= 0.0076) and high Ki‐67 index (*p *= 0.0031) (Table 2), suggesting RASSF4 downregulation serve as an indicator of malignant feature of CRC. The correlations of RASSF4 status with other clinical parameters were not significant.

**TABLE 1:** Expression pattern in normal colon issues and CRC issues

Characteristics	Number of patients	RASSF4 low expression	RASSF4 high expression	*p*
Normal	30	4 (13.3%)	26 (86.7%)	0.0100
Tumour	118	45 (38.1%)	73 (61.9%)	

**TABLE 2:** Distribution of RASSF4 status in colorectal cancer according to clinicopathological characteristics

Characteristics	Number of patients	RASSF4 low expression	RASSF4 high expression	*p*
Age				
<60	51	16 (31.4%)	35 (68.6%)	0.1870
≥60	67	29 (43.3%)	38 (56.7%)	
Gender				
Female	51	15 (29.4%)	36 (70.6%)	0.0887
Male	67	30 (44.8%)	37 (55.2%)	
TNM stage				
Ⅰ+Ⅱ	73	21 (28.8%)	52 (71.2%)	0.0076
Ⅲ+Ⅳ	45	24 (53.3%)	21 (46.7%)	
Tumour status				
T1 T2	34	6 (17.6%)	28 (82.4%)	0.0036
T3 T4	84	39 (46.4%)	45 (53.6%)	
Nodal status				
Negative	73	21 (28.8%)	52 (71.2%)	0.0076
Positive	45	24 (53.3%)	21 (46.7%)	
Differentiation				
Poor	36	16 (44.4%)	20 (55.6%)	0.6381
Moderate	70	25 (35.7%)	45 (64.3%)	
Well	12	4 (33.3%)	8 (66.7%)	
Ki‐67/MIB‐1				
Low expression	49	11 (22.4%)	38 (77.6%)	0.0031
High expression	69	34 (49.3%)	35 (50.7%)	

The Cancer Genome Atlas (TCGA) colorectal cancer cohort revealed that the expression of RASSF4 in colorectal cancer tissues was significantly lower than that in normal colon tissues (Figure 1E). TCGA analysis also indicated that RASSF4 expression was lower in CRCs with nodal metastasis (*p *= 0.0205, Figure 1F), distal metastasis (*p *= 0.0025, Figure 1G) and higher pathological stage (*p *= 0.0095, Figure 1H). In addition, analysis of 41 cases of paired tumour/normal samples showed that RASSF4 was lower in CRC tissues compared with corresponding adjacent normal colon tissues (Figure 1I,J, Wilcoxon matched‐pairs signed‐rank test, *p *< 0.05). The above data suggested that RASSF4 expression was decreased in human CRC.

In addition, we checked RASSF4 protein expression in five colorectal cancer tissues and paired adjacent normal tissues by Western blot. RASSF4 protein was obviously decreased in two out of five of paired cases (case1 and case4) (Figure 1K).

### 3.2 RASSF4 inhibits CRC cell proliferation and cell cycle

**FIGURE 2:**
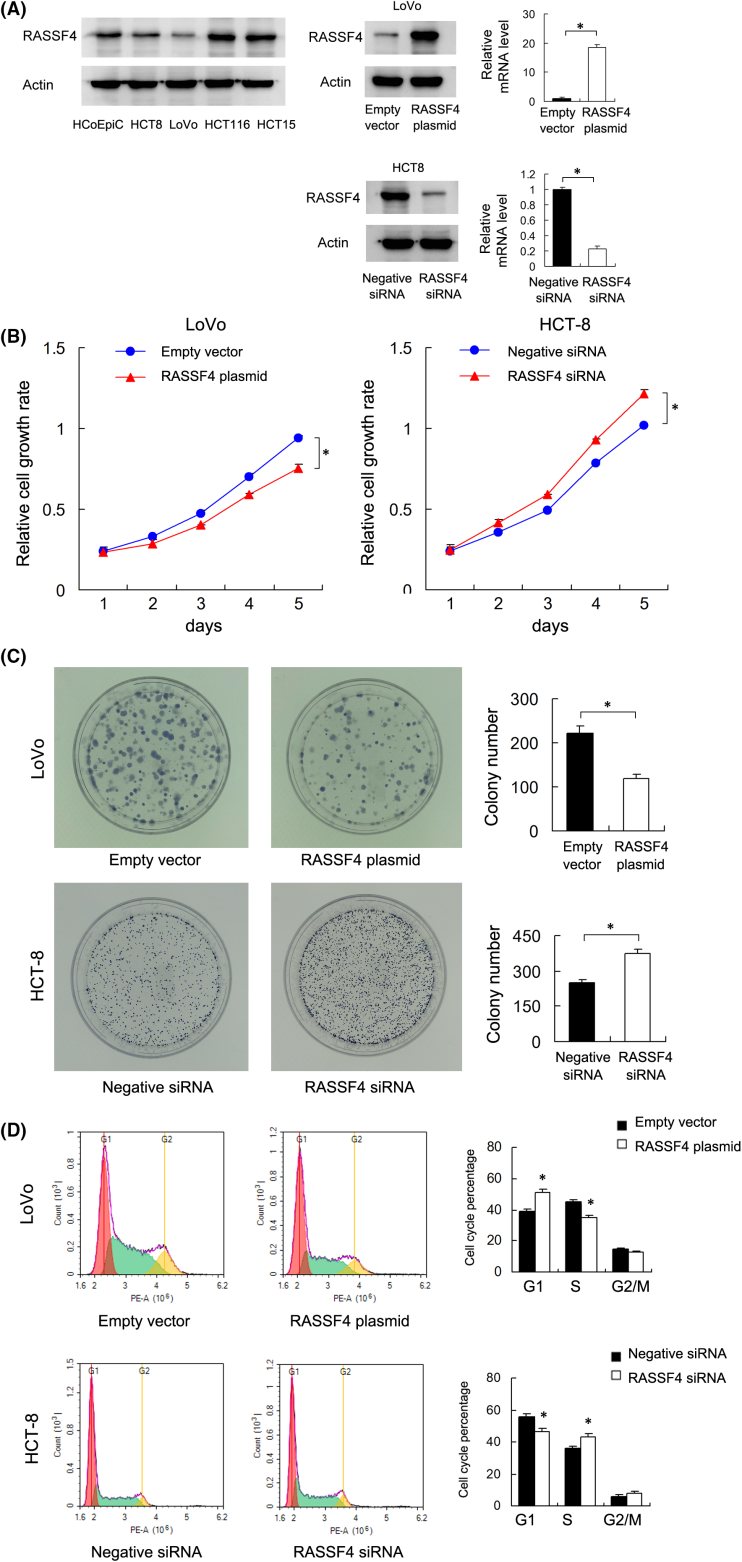
RASSF4 negatively regulates CRC cell proliferation. (A) Western blot analysis in CRC cell lines HCT8, LoVo, HCT116, HCT15 and normal colon epithelial cell line HCO‐Epic. RT‐qPCR and Western blot confirmed the effect of plasmid and siRNA. (B) CCK‐8 demonstrated that RASSF4 overexpression decreased the LoVo cell line's growth speed, while RASSF4 siRNA knockdown increased the cell growth speed in the HCT‐8 cell line. (C) Colony formation assays demonstrated that RASSF4 overexpression decreased colony counts in the LoVo cell line. RASSF4 siRNA knockdown increased colony counts in the HCT‐8 cell line. (D) Flow cytometry demonstrated that ectopic RASSF4 expression decreased the percentage of S phase in the LoVo cell line. RASSF4 knockdown increased the S phase percentage of HCT‐8 cells. *p < 0.05

We first examined RASSF4 protein in four colorectal cancer cell lines (HCT8, LoVo, HCT116 and HCT15) and normal colon epithelial cell line HCO‐Epic. We found relatively lower level of RASSF4 in LoVo cells compared with other cell lines including normal HCO‐Epic cell line (Figure 2A). Thus, we selected LoVo for RASSF4 transfection and HCT‐8 for siRNA knockdown. RT‐qPCR and Western blot were performed to confirm the transfection efficiency of plasmid and siRNA (Figure 2A). Next, we investigated the effect of RASSF4 on CRC cell growth. CCK‐8 analysis showed that RASSF4 transfection inhibited LoVo cell growth rate, while RASSF4 knockdown upregulated HCT‐8 cell growth rate (Figure 2B). Colony formation assay showed that the ectopic expression of RASSF4 reduced LoVo cell colony formation ability while RASSF4 knockdown increased colony formation ability in HCT‐8 cells (Figure 2C).

Cell cycle analysis showed that RASSF4 overexpression downregulated S phase percentage while upregulated G1 phase percentage in LoVo cell line. RASSF4 knockdown in HCT‐8 cell line showed the opposite effects (Figure 2D). The above results indicated that RASSF4 inhibited CRC cell growth by regulating cell cycle progression.

### 3.3 RASSF4 enhances 5‐FU sensitivity and mitochondrial membrane potential

**FIGURE 3:**
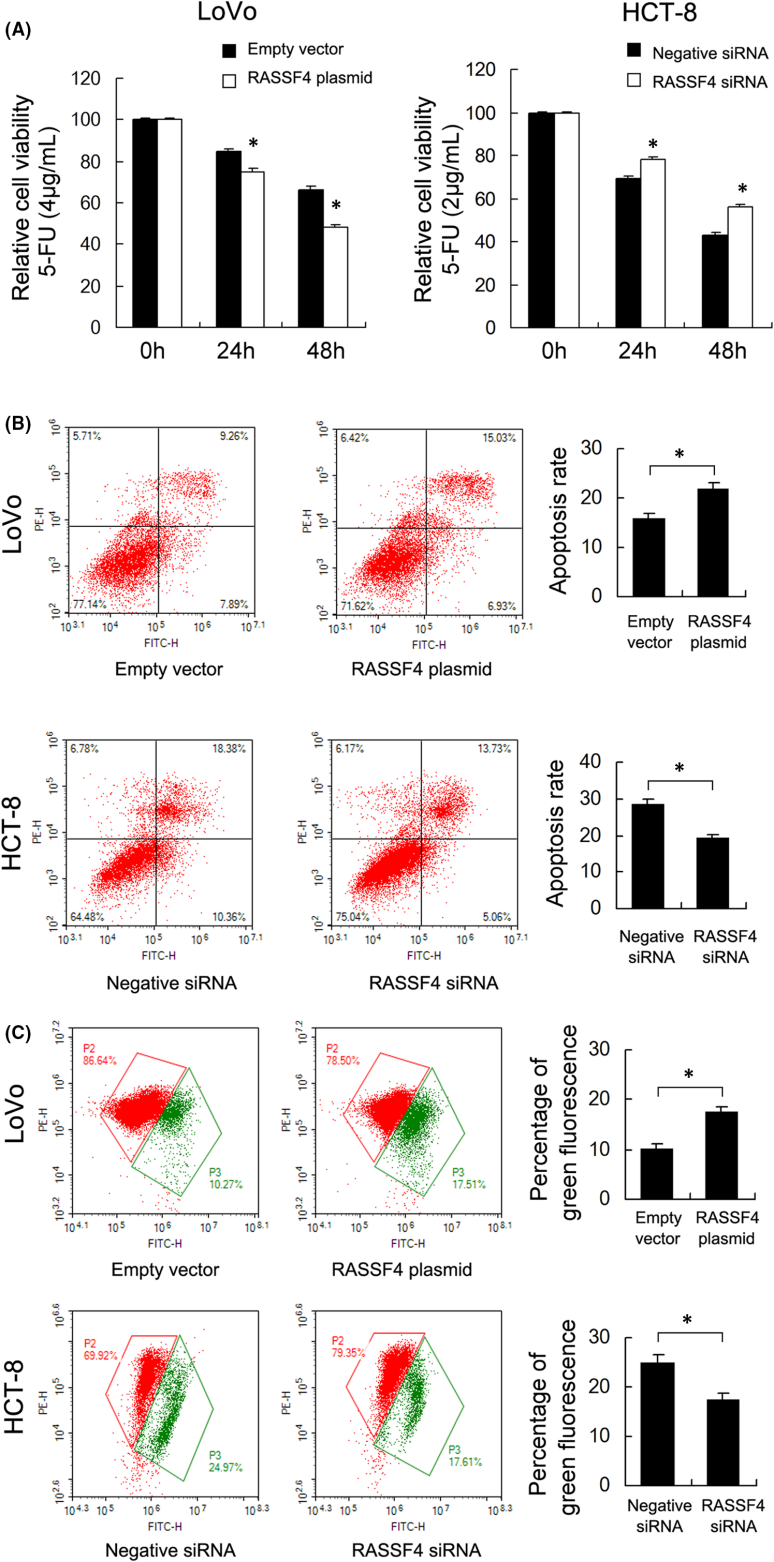
RASSF4 regulates 5‐FU sensitivity in CRC cells. (A) CCK‐8 assays showed that ectopic RASSF4 expression decreased cell viability in LoVo cells treated with 4 μg/ml 5‐FU, while RASSF4 depletion increased cell viability in HCT‐8 cells treated with 2 μg/ml 5‐FU. (B) Annexin V/PI analysis demonstrated that RASSF4 overexpression significantly increased apoptosis rate in LoVo cells treated with 5‐FU, while RASSF4 siRNA depletion downregulated apoptosis rate in HCT‐8 cells treated with 5‐FU. (C) JC‐1 staining showed that ectopic RASSF4 expression in LoVo cells upregulated green percentage, indicating a downregulating effect of RASSF4 on Δψm. RASSF4 siRNA treatment showed the opposite effect in HCT‐8 cells. *p < 0.05

5‐Fluorouracil (5‐FU) remains the first‐line treatment for colorectal cancer (CRC). We examined the change of 5‐FU sensitivity after RASSF4 overexpression/knockdown. A solution of 4 μg/ml 5‐FU was used to treat LoVo cells and 2 μg/ml 5‐FU was used to treat HCT‐8 cells. CCK‐8 assay showed that ectopic expression RASSF4 in LoVo cells upregulated 5‐FU inhibition rate, while RASSF4 knockdown increased HCT‐8 cell resistance to 5‐FU (Figure 3A). Annexin V/PI staining was used to examine change of apoptosis rate. RASSF4 overexpression significantly increased apoptosis rate in LoVo cells treated with 5‐FU, while RASSF4 siRNA depletion downregulated apoptosis rate in HCT‐8 cells treated with 5‐FU (Figure 3B). The above results indicated that RASSF4 increased 5‐FU sensitivity in CRC cells.

Mitochondrial membrane potential plays a crucial role in cell survival as well as chemical resistance. We examined change of mitochondrial membrane potential (Δψm) using JC‐1 staining in RASSF4 overexpressed and depleted CRC cells. JC‐1 dye exhibits red fluorescence during high Δψm and green fluorescence in cells with low Δψm. As shown in Figure 3C, in CRC cells treated with 5‐FU, ectopic RASSF4 expression in LoVo cells upregulated green percentage, indicating a downregulating effect of RASSF4 on Δψm. RASSF4 siRNA treatment showed the opposite effect in HCT‐8 cells (Figure 3C).

### 3.4 RASSF4 regulates YAP in CRC cells

**FIGURE 4:**
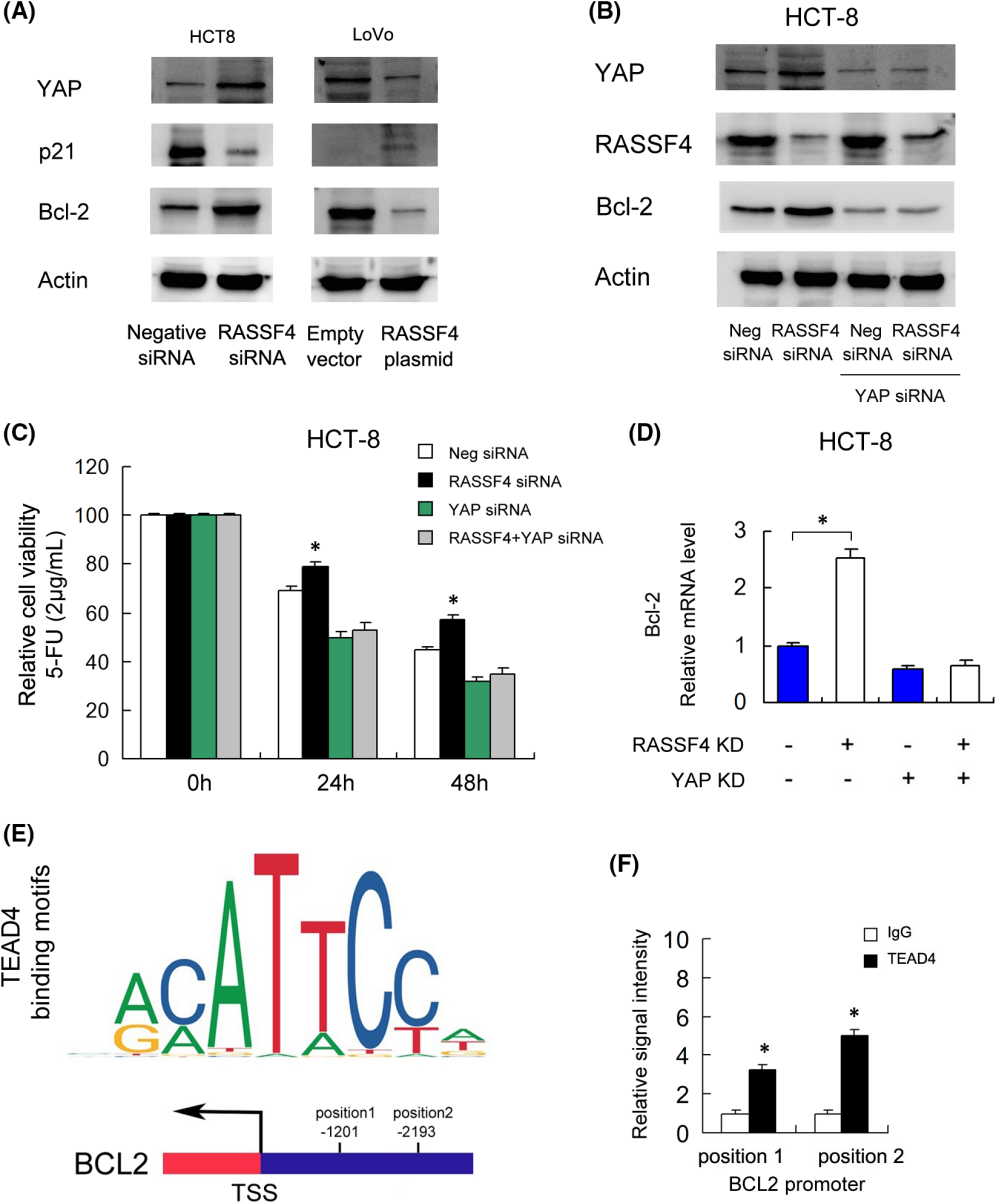
RASSF4 regulates Bcl‐2 and 5‐FU sensitivity through YAP. (A) Western blot demonstrated that RASSF4 overexpression decreased the protein expression of YAP, Bcl‐2 and increased p21. RASSF4 knockdown increased the protein expression of YAP, Bcl‐2 and decreased p21. (B) YAP siRNA efficiently downregulated YAP protein. YAP knockdown also downregulated Bcl‐2 protein. In YAP‐depleted cells, the effects of RASSF4 siRNA on Bcl‐2 were largely abolished. (C) CCK‐8 assay showed that in HCT‐8 cells treated with YAP siRNA, the change of 5‐FU sensitivity induced by RASSF4 knockdown was significantly reduced. (D) RT‐qPCR showed that YAP knockdown significantly suppressed mRNA expression of Bcl‐2. YAP depletion also ameliorated the effect of RASSF4 siRNA on Bcl‐2. (E) Prediction of potential TEAD4 binding motifs using JASPAR database. (F) Chromatin immunoprecipitation (ChIP) assay demonstrated that TEAD4 could bind to the Bcl‐2 promoter regions. *p < 0.05

Since RASSF family proteins have been reported to influence the activity of the Hippo pathway, we checked the changes of YAP protein expression after RASSF4 overexpression/knockdown, which serves as a Hippo effector. Western blot showed that RASSF4 overexpression decreased the expression of YAP, Bcl‐2 and increased p21 in LoVo cell line (Figure 4A). RASSF4 depletion upregulated YAP, Bcl‐2 and downregulated p21 expression in HCT‐8 cell line.

### 3.5 RASSF4 regulates Bcl‐2 and 5‐FU sensitivity through YAP

To determine whether YAP was responsible for the change of Bcl‐2 regulated by RASSF4. We examined the change of YAP and 5‐FU sensitivity after RASSF4 knockdown in YAP‐depleted HCT‐8 cells. As shown in Figure 4B, YAP siRNA efficiently downregulated YAP protein. YAP knockdown also downregulated Bcl‐2 protein. In addition, YAP depletion ameliorated Bcl‐2 upregulation induced by RASSF4 siRNA, indicating the effect of RASSF4 upon Bcl‐2 is in a YAP‐dependent manner (Figure 4B).

CCK‐8 assay showed that in HCT‐8 cells treated with YAP siRNA, the change of 5‐FU sensitivity induced by RASSF4 knockdown was significantly reduced (Figure 4C). RT‐qPCR showed that YAP knockdown significantly suppressed mRNA expression of Bcl‐2. YAP depletion also ameliorated the effect of RASSF4 siRNA on Bcl‐2 (Figure 4D).

YAP is a co‐activator of TEAD4 transcription factor directly interacting with the promoter regions of Hippo targets. We then examined if TEAD4 regulated Bcl‐2 through its promoter. We used JASPAR to predict the binding motifs and 2 potential binding sites (positions1: −1201; position2: −2193) (Figure 4E). Chromatin immunoprecipitation assay (ChIP) demonstrated that TEAD4 could interact with Bcl‐2 promoter regions (Figure 4F). The above results indicated that RASSF4 could regulate Bcl‐2 through YAP in CRC.

## 4 DISCUSSION

RASSF4 downregulation has been implicated in several human cancers such as nasopharyngeal carcinoma, gastric adenocarcinoma, multiple myeloma and lung cancer.^9^, ^10^, ^11^, ^12^ However, its expression pattern and clinical significance in human CRC have not been defined. Our data demonstrated that RASSF4 protein expression was downregulated in 38.2% of CRC specimens, which was correlated with TNM stage, nodal status and Ki‐67 proliferation index. Our results were further supported by TCGA data analysis, showing RASSF4 downregulation in CRC cancers and its negative correlation with pathological stage. Our results indicated that loss of RASSF4 could serve as a potential biomarker for malignant progression of CRC.

RASSF4 overexpression has been reported to induce 293T cell apoptosis and inhibit growth in cancer cell line A549 and MCF7.^8^ To confirm its role in CRC cells, we picked LoVo cell line with low endogenous expression for transfection and HCT‐8 cell line for siRNA knockdown. Our results showed that ectopically expressed RASSF4 in LoVo cells reduced proliferation, colony formation and cell cycle progression, suggesting RASSF4 serves as a negative regulator of CRC cell growth. Accordingly, we found that RASSF4 overexpression upregulated p21 while RASSF4 knockdown downregulated p21. p21 functions as an inhibitor of cell cycle, which inhibits kinase activity and block progression through G1/S. Loss of p21 was found in various human cancer which indicated uncontrolled growth of cancer cells.^15^, ^16^ Thus, RASSF4 might regulate CRC proliferation through regulation of cell cycle and p21 protein.

Development of chemoresistance is one important causes of the poor prognosis of CRC patient. We investigated the potential role of RASSF4 on 5‐FU sensitivity. Our results revealed that overexpression of RASSF4 increased 5‐FU sensitivity and apoptosis rate of CRC cells. Since chemosensitivity and apoptosis are closely related to mitochondrial function,^17^, ^18^, ^19^ we checked change of mitochondrial membrane potential (Δψm) using JC‐1 staining assay. Our data showed that RASSF4 downregulated Δψm while RASSF4 knockdown upregulated Δψm in CRC cells treated with 5‐FU, suggesting loss of RASSF4 might increase mitochondrial function in CRC cells.

By the fact that RASSF4 regulated chemosensitivity and mitochondrial function in CRC cells, our data further showed that RASSF4 downregulated Bcl‐2 expression. Bcl‐2 family proteins are considered as one of the major regulators of mitochondrial homeostasis and apoptosis.^20^, ^21^ Downregulation of Bcl‐2 function and expression leads to increased sensitivity to apoptosis.^22^ Clinically, Bcl‐2 inhibitors, such as venetoclax and navitoclax, were shown to selectively induce apoptosis in malignant cells.^23^ Overexpression of Bcl‐2 contributes to the development of resistance to chemotherapy.^24^ Breast cancer patients with high Bcl‐2 expression had a poor response to chemotherapy compared with those who had less Bcl‐2 expression.^25^ RASSF proteins have been reported to negatively regulate YAP expression and nuclear translocation,^26^ which is a transcription co‐activator interacting with TEAD4, a transcription factor controls Hippo target genes, including Bcl‐2.^27^ Previous studies have reported that increased expression of YAP contributes to cancer progression, including CRC. More importantly, YAP can inhibit autophagy and promote progression of colorectal cancer via upregulating Bcl‐2 expression, while autophagy‐related pathways are enriched and activated in CRC patients and can induce cancer chemoresistance.^24^, ^28^ In our study, we also checked YAP and found RASSF4 downregulated its expression in CRC cells. Using YAP siRNA, we demonstrated that YAP mediated the effect of RASSF4 on Bcl‐2 in HCT‐8 cell line. In addition, our ChIP data supported the fact that TEAD4 could bind to the Bcl‐2 promoter region, thus demonstrating a strong link among RASSF4, YAP/TEAD4 and Bcl‐2.

In conclusion, the current study showed that loss of RASSF4 indicated malignant phenotype in CRC. RASSF4 could inhibit CRC cell proliferation and increase chemosensitivity possibly by decreasing Bcl‐2 expression through regulation of YAP. However, CRC is a multistep ‘genetic’ disorder, the mechanism is extremely complex. Numerous reports have also suggested that the transcription factors nuclear factor‐kB (NF‐kB) and activator protein‐1 (AP‐1) can regulate the expression of Bcl‐2 leading to the transition of benign carcinomas towards malignant phenotype.^29^ Whether the biological role of RASSF4 in colon cancer involves NF‐kB or AP‐1 and whether it plays a protective role in vivo requires further investigation.

## AUTHOR CONTRIBUTION

**Yong Han:** Conceptualization (equal); Data curation (equal); Formal analysis (equal); Funding acquisition (lead); Methodology (equal); Project administration (equal); Supervision (equal); Writing – original draft (equal); Writing – review & editing (equal). **Xiaotang Zhang:** Data curation (equal); Investigation (equal); Methodology (equal); Writing – original draft (equal). **Minmin Guan:** Data curation (equal); Formal analysis (equal); Investigation (equal); Methodology (equal); Writing – original draft (equal). **Cheng Huo:** Investigation (equal); Methodology (equal); Resources (equal); Writing – original draft (equal). **Chunlin Yu:** Data curation (equal); Formal analysis (equal); Investigation (equal); Methodology (equal). **Bin Hu:** Conceptualization (equal); Data curation (equal); Investigation (equal); Methodology (equal); Validation (equal). **Jianjun Cai:** Resources (equal); Supervision (equal); Writing – review & editing (equal).

## CONFLICT OF INTEREST

We declare that we have no conflict of interest.

## Data Availability

The data that support the findings of this study are available from the corresponding author upon reasonable request.
